# Associations Between Genetic Variants in the 17q21 Locus, *CDHR3* and Childhood Asthma

**DOI:** 10.3390/children13091188

**Published:** 2026-09-03

**Authors:** Anastasia Filiou, Angela Hoyer, Idun Holmdahl, Marianne van Hage, Björn Nordlund, Gunilla Hedlin, Jon R. Konradsen, Cilla Söderhäll

**Affiliations:** 1 Department of Women’s and Children’s Health, Karolinska Institutet, 171 77 Stockholm, Sweden; angela.hoyer@ki.se (A.H.); idun.holmdahl@ki.se (I.H.); bjorn.nordlund@ki.se (B.N.); gunilla.hedlin@ki.se (G.H.); jon.konradsen@ki.se (J.R.K.); cilla.soderhall@ki.se (C.S.); 2 Astrid Lindgren’s Children’s Hospital, Karolinska University Hospital, 171 76 Stockholm, Sweden; 3 Sachsska Children’s Hospital, 118 83 Stockholm, Sweden; 4 Department of Clinical Science and Education, Södersjukhuset, 118 83 Stockholm, Sweden; 5 Department of Medicine Solna, Division of Immunology and Allergy, Karolinska Institutet and Karolinska University Hospital, 171 77 Stockholm, Sweden; marianne.van.hage@ki.se; 6 Center for Molecular Medicine, Karolinska Institutet, 171 76 Stockholm, Sweden

**Keywords:** asthma, genetic variants, the 17q21 locus

## Abstract

**Highlights:**

**What are the main findings?**

- Genetic variants in the 17q21 locus are strongly associated with asthma at 7 years in children with a history of preschool wheeze.
- Rhinovirus-induced wheeze at preschool age and rs6967330 (*CDHR3*, coding for Rhinovirus C receptor) are associated with asthma at 7 years among children with specific genotypes in the 17q21 locus.

**What are the implications of the main findings?**

- Rhinovirus-induced wheeze is a significant marker of underlying susceptibility.
- There might be differential patterns of association between 17q21 variants and asthma according to rhinovirus exposure, warranting further investigation in larger cohorts.

**Abstract:**

**Background:** Previously identified asthma-susceptibility genes account for a small part of asthma heritability and their role in asthma pathogenesis is unclear. We explored associations between genetic variants in the 17q21 locus, *CDHR3* (cadherin-related family member 3)*,* coding a receptor for Rhinovirus-C, preschool wheeze and asthma at 7 years. **Methods:** Four genetic variants in the 17q21 locus (rs8076131, rs12603332, rs8079416, rs3859192) and rs6967330 in *CDHR3* were studied regarding associations with preschool wheeze and asthma at 7 years. We compared 125 cases, enrolled during an acute wheezing episode, with 96 healthy controls at preschool age (6–45 months). A total of 99 of 125 children attended the follow-up at 7 years old. Cases with asthma (N = 68) and without asthma (N = 31) at 7 years old were compared regarding genetic variants and other clinical parameters. **Results:** Rs8076131 (AA vs. GG) was associated with preschool wheeze (OR 3.50, *p* = 0.001), and asthma at 7 years (OR 8.55, *p* = 0.002). Rs12603332 (CC vs. TT) was related to asthma at 7 years irrespective of rhinovirus infection at inclusion or current signs of airborne allergy (aOR 7.17, *p* = 0.016). The association of rs6967330 with asthma was restricted to children with specific genotypes in the 17q21 locus; rs8076131-AA **(***p* = 0.028)**,** rs8079416-CC (*p* = 0.006), and rs3859192-TT (*p* = 0.042). Rhinovirus infection at inclusion was significantly related to asthma exclusively in homozygotes rs8079416-CC (*p* = 0.032) and rs3859192-TT (*p* = 0.027). **Conclusions:** Our results highlight the impact of asthma heritability by reporting strong associations between the 17q21 locus and asthma in a high-risk cohort. The association of rs6967330 in *CDHR3* and early-life rhinovirus infection with asthma at school age might be dependent on specific genotypes in the 17q21 locus.

## 1. Introduction

Asthma is a multifactorial disease, with heterogeneous clinical presentation and pathogenesis. Fifty percent of individuals with asthma report wheeze during the first 6 years of life [1]. Conversely, 40% of wheezy children at preschool age continue to wheeze later in childhood while the remaining 60% outgrow their symptoms [1]. Multiple factors are shown to be associated with persistent asthma in preschool wheezers: genetic predisposition, atopic disease, tobacco smoke exposure, vitamin D insufficiency, and respiratory tract infections in early life [2,3,4,5,6].

Although several genetic variants are shown to be associated with childhood asthma, their effect sizes and contribution to asthma heritability are small [7]. This could be explained by interactions between environmental and genetic factors [3,8,9], gene–gene interactions as well as the age-dependent manifestation and the phenotypical diversity of asthma [10]. Genetics seem to contribute to asthma to a greater extent in childhood than in adulthood, which is evidenced by the reported inverse relationship between estimates of heredity and age of asthma onset [11].

Genome-wide association studies (GWAS) have revealed several asthma-susceptibility genetic variants in the 17q21 locus. The associations between genetic variants and asthma in this locus might be influenced by the age of onset [12]. Some variants of orosomucoid-like 3 (*ORMDL3*) and gasdermin B (*GSDMB*) genes in the 17q21 locus have been associated exclusively with childhood-onset asthma [3,13]. However, the exact physiologic role of ORMDL3 and GSDMB in asthma pathogenesis is not fully understood yet [14].

The effect of some genetic variants in 17q21 on the risk of asthma is evident among children with Rhinovirus (RV)-induced wheeze at preschool age [15]. A genetic variant in Cadherin-related family member 3 (*CDHR3*), coding a receptor for RV-C, and highly expressed in airway epithelium, has been shown to be associated with RV-C illness, early-life wheezing as well as asthma at school age [16,17,18]. Nevertheless, the contribution of *CDHR3*-related genetic risk to asthma persistence is still ambiguous [19].

Given the phenotypical heterogeneity of asthma and the complexity of its pathogenesis, investigating risk factors in homogeneous groups of children with wheezing illness or asthma might shed light on specific etiological pathways [20]. In this study, 125 children were included during an episode of acute preschool wheeze and followed prospectively to age 7 years. We selected a panel of genetic variants in the 17q21 locus, based on published GWAS [3,13], and investigated them in our cohort using a candidate gene approach. Our aim was to study the association between these genetic variants in the 17q21 locus as well as *CDHR3* with preschool wheeze (PW) and asthma at school age.

## 2. Materials and Methods

### 2.1. Study Base and Design

The study population included in this study is a subset of the ‘Gene Expression in Wheezing and Asthmatic Children’ (GEWAC) cohort. ‘Gene Expression in Wheezing and Asthmatic Children’ (GEWAC) is a cohort of 156 preschool wheezers (cases) and 102 age-matched healthy controls. The cases (age 6–48 months) were enrolled at the pediatric emergency ward at Astrid Lindgren’s Children’s Hospital, Stockholm, Sweden, between 2008 and 2012 when seeking care for acute wheeze. Exclusion criteria were prematurity (birth before 36 gestational weeks), other chronic diseases or any simultaneous complications such as sepsis, bacterial pneumonia or diabetes at the time point of inclusion. Enrollment took place from October 2008 until June 2012. The cases were assessed at inclusion, and they were invited to follow-ups after 2–3 months, one year and at age 7 years [21]. Blood samples were drawn at the emergency visit and at later revisits. A large proportion of cases (81%) needed hospitalization due to severe symptoms, 52% had a diagnosis of asthma already at the first follow-up and 70.8% was diagnosed with asthma at age 7 years [21], which supports the high-risk nature of the GEWAC cohort. The controls were enrolled at the Surgical Day-care Ward, Astrid Lindgren Children’s Hospital, where they had minor surgery performed, and blood was drawn at the same time as an intravenous line was inserted prior to surgery and anesthesia. Exclusion criteria were prematurity (birth before 36 gestational weeks) and/or a history of bronchial obstruction/asthma or known sensitization to airborne allergens [4]. Inclusion and exclusion criteria are listed in Table 1.

**Figure 1:**
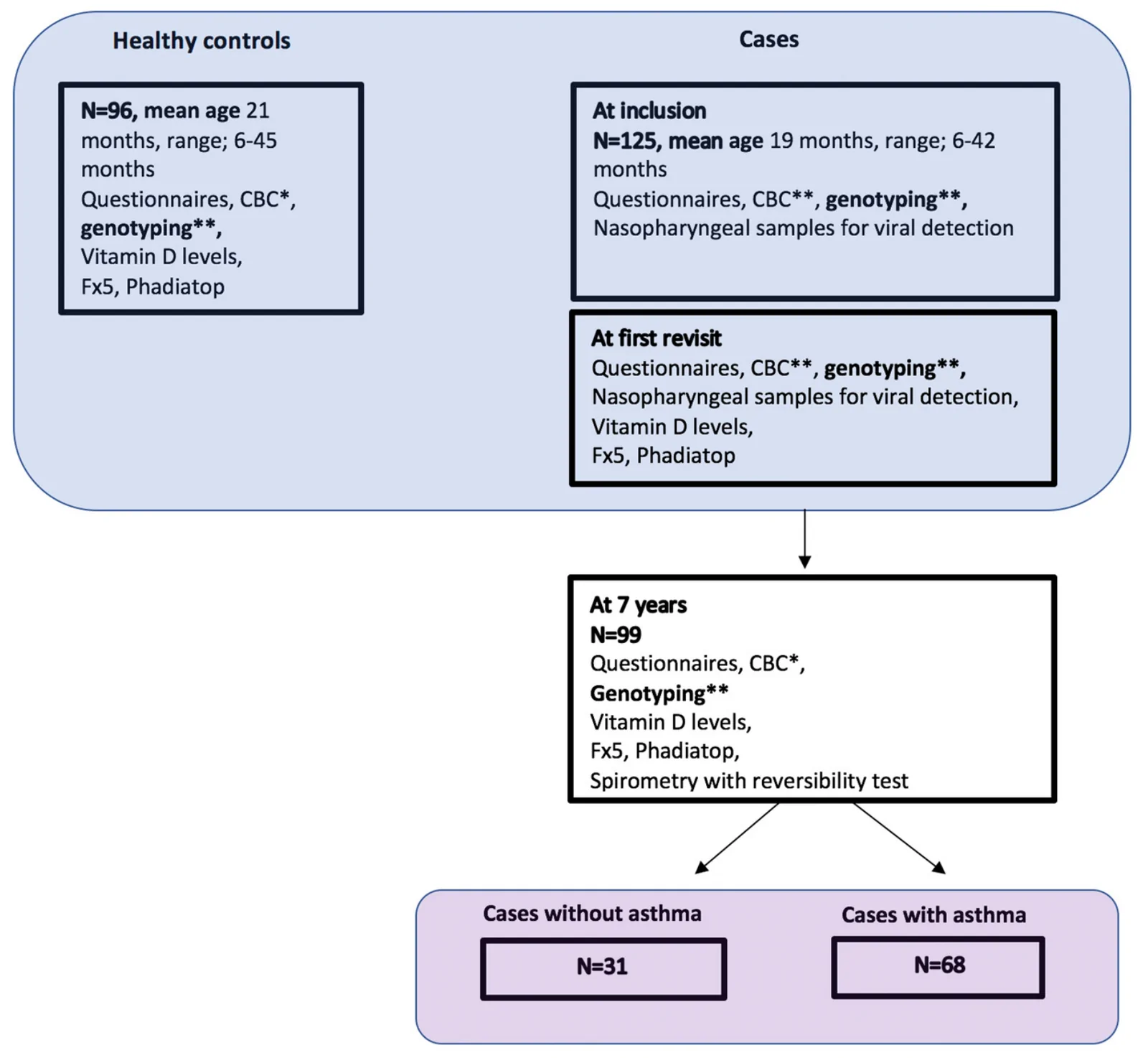
Flow-chart of the GEWAC population included in the current study; The coloured boxes represent the comparisons performed between pairs of groups. Blue indicates the groups and assessments at inclusion, whereas purple indicates asthma status among cases at 7 years of age. * CBC complete blood count, ** Genotypes for studied genetic variants in the 17q21 locus included in Figure 2. All children included in the study have available genotype(s) for at least one of the studied genetic variants. Phadiatop; allergen-specific IgE against common airborne allergens birch, timothy, mug worth, cat, dog, horse, molds (Cladosporium herbarum), dust mites (Dermatophagoides pteronyssinus, Dermatophagoides farina), Fx5; allergen-specific IgE against common food allergens cow’s milk, egg white, soy bean, peanut, cod fish and wheat.

The study population of this study consisted of 125 cases and 96 controls with genotypes available for at least one of the studied genetic variants in the 17q21 locus (Figure 1, Table S1). A total of 99 of 125 children had available genotype data and information on asthma status at the follow-up at 7 years old and were included in the analyses of genetic associations with asthma at 7 years. We compared cases with asthma at 7 years (68/99) and cases without asthma at 7 years (31/99) with respect to all studied genetic variants.

### 2.2. Data Sources

Standardized questionnaires regarding ethnicity, lifestyle, family history of asthma and allergies, breastfeeding, exposure to tobacco smoke, pets and previous infections as well as occurrence of atopic and asthma symptoms and use of asthma medication was filled out by the legal guardians at all visits. The study physician interviewed the guardians and filled out a standardized questionnaire concerning the number of days the child had suffered from asthma symptoms at home (cough, wheeze and/or shortness of breath), use of medication (inhaled corticosteroids (ICS), leukotriene receptor antagonist (LTRA) or oral corticosteroids), as well as any emergency visits between the acute and follow-up visits. The reported number of emergency visits was checked against medical journals, and the guardians’ personal diaries of days spent away from work when the child had respiratory symptoms.

### 2.3. Asthma Definition

The definition of asthma at 7 years of age was based on Global Initiative for Asthma (GINA) guidelines [22] and included one mandatory criterion (a diagnosis of asthma by the study doctor, a pediatric allergist) and one out of three additional criteria: lower respiratory symptoms, medication for treatment of wheeze for 5 days or longer in the preceding 12 months (ICS or LTRA), or airway reversibility >12% after the use of bronchodilator with salbutamol, as previously described [21]. Lower respiratory symptoms were defined as cough, shortness of breath, or nocturnal awakening caused by wheeze for 5 days or longer in the preceding 12 months. All children that fulfilled the compulsory and at least one of the additional criteria (symptoms, medication, or reversibility) were classified as having asthma. Children with a doctor’s diagnosis of asthma were diagnosed by different doctors, before or after inclusion in the study according to national guidelines. Spirometry and airway reversibility were tested using the Medikro Pro spirometer (Medikro Oy, Kuopio, Finland). Reversibility was evaluated 15 min after inhalation with 400 micrograms salbutamol using a spacer. The patient’s technique and quality of the spirometry were evaluated according to international guidelines, and if the test criteria were not fulfilled, the test result was not included in the analyses [21].

### 2.4. Sample Collection and Laboratory Analyses

Nasal swab tests for the presence of virus were obtained from the cases at the emergency department and again at the 3-month follow-up, and transported within two hours after collection in a standardized virus medium (Sigma-Virocult, Medical Wire & Equipment Co Ltd., Corsham, Wiltshire, UK). The swabs were analyzed according to standard procedures by real-time PCR using a 15 virus platform developed in 2007 (including Adenovirus, Bocavirus, Coronavirus (229E, HKU1, NL63), Influenza A, Influenza A/H1N1 and Influenza B, Parainfluenza virus (1,2,3), Metapneumovirus, Enterovirus, Rhinovirus and Respiratory Syncytial virus) [23], and then stored in the biobank at the Department of Clinical Microbiology, Karolinska University Hospital.

Levels of Vitamin D 25(OH)D were analyzed at the Karolinska University Hospital Laboratory using a standard procedure involving direct, competitive chemiluminescence analysis (CLIA, DiaSorin Inc, Stillwater, MN, USA) for the blood samples from the 3-month revisit, and using competitive immunoassay (Roche Diagnostics Vitamin D total assay) for the blood samples at the follow-up at 7 years old. Allergen-specific IgE against common airborne allergens (Phadiatop; birch, timothy, mug worth, cat, dog, horse, molds (Cladosporium herbarum), house dust mites (Dermatophagoides pteronyssinus, Dermatophagoides farina)) and food allergens (Fx5; cow’s milk, egg white, soy bean, peanut, codfish and wheat) (Thermo Fisher Scientific, Uppsala, Sweden), were analyzed in blood samples at the first follow-up visit and at 7 years. Vitamin D insufficiency was defined as 25(OH)D 50 to 75 nmol/L and allergic sensitization as IgE ≥ 0.35 kU_A_/L against Phadiatop and/or Fx5.

DNA was extracted from whole blood using standard methods. The 13 most frequently studied genetic variants in the 17q21 locus and *CDHR3* (rs6967330) were selected (Table S2) based on major GWAS [3,13] and genotyped using TaqMan allelic discrimination according to the manufacturer’s protocol (Thermo Fisher Scientific, Uppsala, Sweden). DNA quality and quantity was assessed using Nanodrop 8000 (ThermoFisher Scientific). All TaqMan assays showed satisfactory performance, all with less than 1.5% missing genotypes (genotyping call rate >98.5%). Genotype calling was performed automatically by the genotyping platform and the laboratory staff was blinded to the clinical data.

### 2.5. Statistical Analyses

SPSS version 26 (IBM) was used. An un-paired *t*-test was used to analyze continuous variables with normal distribution and Mann–Whitney or Kruskal–Wallis (nonparametric tests) were applied for skewed continuous variables. We checked for Hardy–Weinberg equilibrium in cases (N = 125) and controls (N = 96) and for linkage disequilibrium (LD, calculation of r^2^) in Haploview. Chi-square test was used to compare the distribution of genotypes and allele frequencies of genetic variants between cases and controls as well as between cases with and without asthma at the same age. Fisher’s exact test was applied when sample sizes were limited.

**Figure 2:**
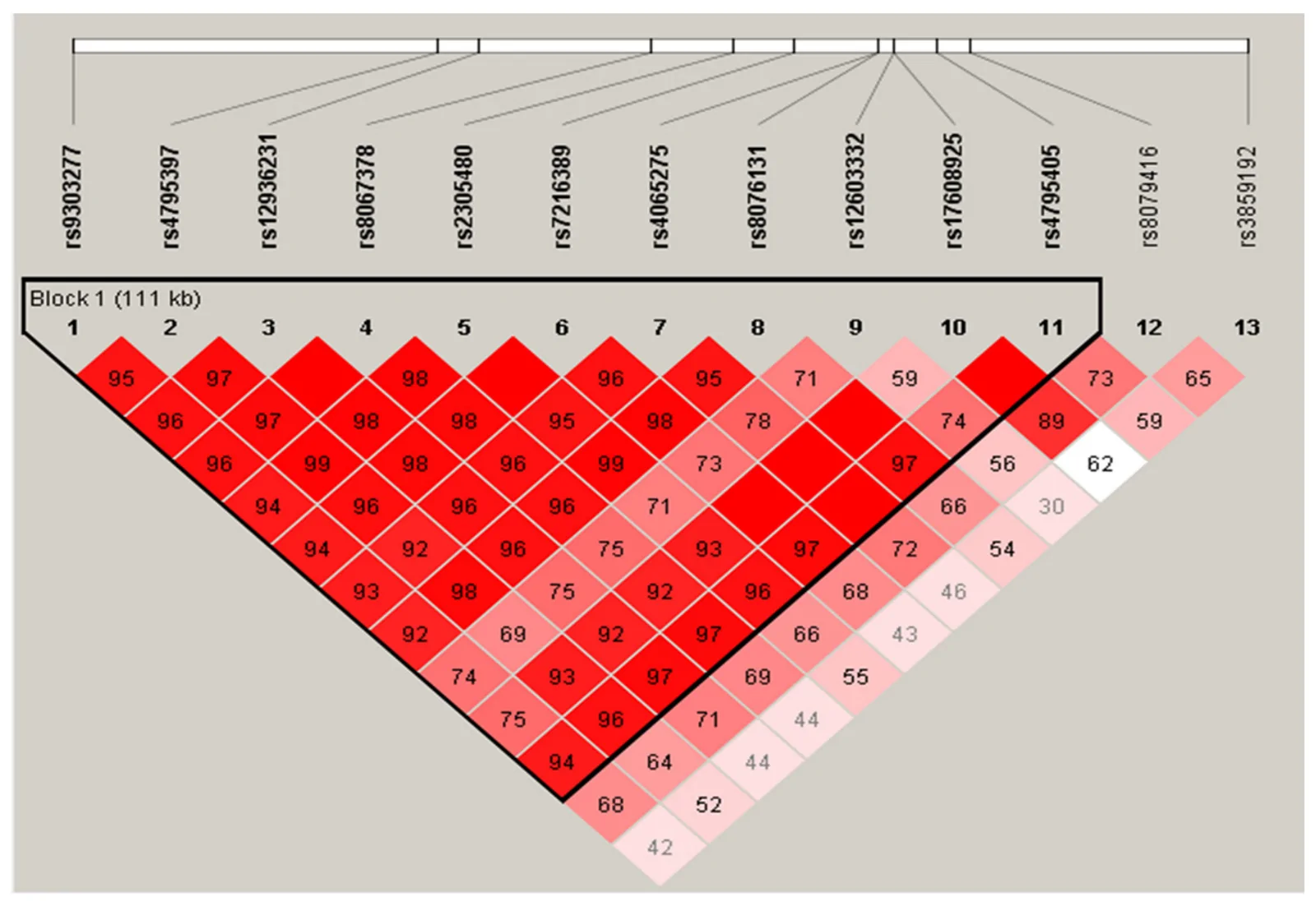
The genetic variants in the 17q21 locus included in the current study are shown, with higher linkage disequilibrium (LD) observed between the first eight genetic variants (from the left). Color intensity represents the strength of LD, with darker red indicating higher pairwise r^2^ values, while numbers within the diamonds indicate the corresponding pairwise r^2^ values.

Thirteen genetic variants within the 17q21 locus were selected a priori for the initial analyses (Table S1, Figure 2), based on previously reported associations with childhood asthma or their location within the 17q21 asthma-susceptibility region, with the aim of capturing genetic variation across the locus. Four variants were subsequently selected for further analyses based on the LD structure of the region and their ability to provide complementary representation of the locus, independently of their observed associations with the study outcomes. These included rs8076131, selected as a proxy for nine highly correlated variants within the high-LD block; rs12603332, which showed the lowest pairwise r^2^ with the other variants within this block; and rs8079416 and rs3859192, which were located in the flanking regions (Figure 2). The maximum pairwise r^2^ among the four selected variants was 0.71.

Logistic regression was used to assess the associations between the genetic variants and preschool wheeze and asthma at 7 years. Variables significantly associated with preschool wheeze or asthma at 7 years in the univariate analyses (Table S2) were included in multivariable logistic regression models to calculate adjusted odds ratios (aORs) for the respective outcomes.

## 3. Results

A higher proportion of cases (N = 125) had heredity for asthma and/or allergy, and eczema at inclusion than controls (N = 96, Table S2). No differences were seen between cases included in the analyses at 7 years (N = 99) and cases that were lost to follow-up (N = 26, Table S3). Forty-nine genotyped controls, not included in this study population, were assessed at 7 years and one of them (N = 1/49) was diagnosed with asthma at this age.

Among 99 children with PW, 68 (68.7%) were diagnosed with asthma at 7 years. Rhinovirus (RV) infection at inclusion and current allergic rhinitis or conjunctivitis at age 7 years were significantly overrepresented among asthmatics (N = 68) compared to non-asthmatics (N = 31) at 7 years (Table S2). Nine genetic variants in the 17q21 locus were associated with both preschool wheeze and asthma at 7 years. Rs12603332 was associated with asthma at 7 years but not with PW. Rs17608925 and rs8079416 were related to PW but not to asthma at 7 years. Rs3859192 was neither associated with PW nor with asthma at 7 years (Table 2).

### 3.1. Associations Between Genetic Variants in the 17Q21 Locus with Preschool Wheeze

The associations of rs8076131 (AA vs. GG; OR= 3.50, 95% CI 1.60–7.66, *p* = 0.001), and rs8079416 (CC vs. TT; OR = 2.42, 95% CI 1.12–5.23, *p* = 0.024) with PW (Table 2) remained significant after adjustment for other risk factors for PW (Table S2 and Table 3). In the adjusted analysis, increasing numbers of effect alleles in rs8076131 (aOR = 2.36, 95% CI 1.47–3.78, *p* < 0.001) and rs8079416 (aOR = 1.78, 95% CI 1.12–2.84, *p* = 0.015, Table 3) carried by an individual were significantly associated with PW. The rs6967330-A (*CDHR3*) was significantly associated with PW (Table S4).

### 3.2. Associations Between Genetic Variants in the 17Q21 Locus and Asthma at 7 Years

The associations of rs8076131 (AA vs. GG; OR= 8.55, 95% CI 2.18–33.59, *p* = 0.002), and rs12603332 (CC vs. TT; OR= 4.50, 95% CI 1.27–15.90, *p* = 0.016) with asthma at 7 years (Table 2) remained significant after adjustment for RV infection at inclusion and current allergic rhinitis or conjunctivitis at 7 years (Table 3). When stratifying by genotype, RV infection at inclusion significantly increased the risk of asthma at 7 years in cases with rs8079416-CC (*p* = 0.032) or rs3859192-TT (*p* = 0.027).

**Figure 3:**
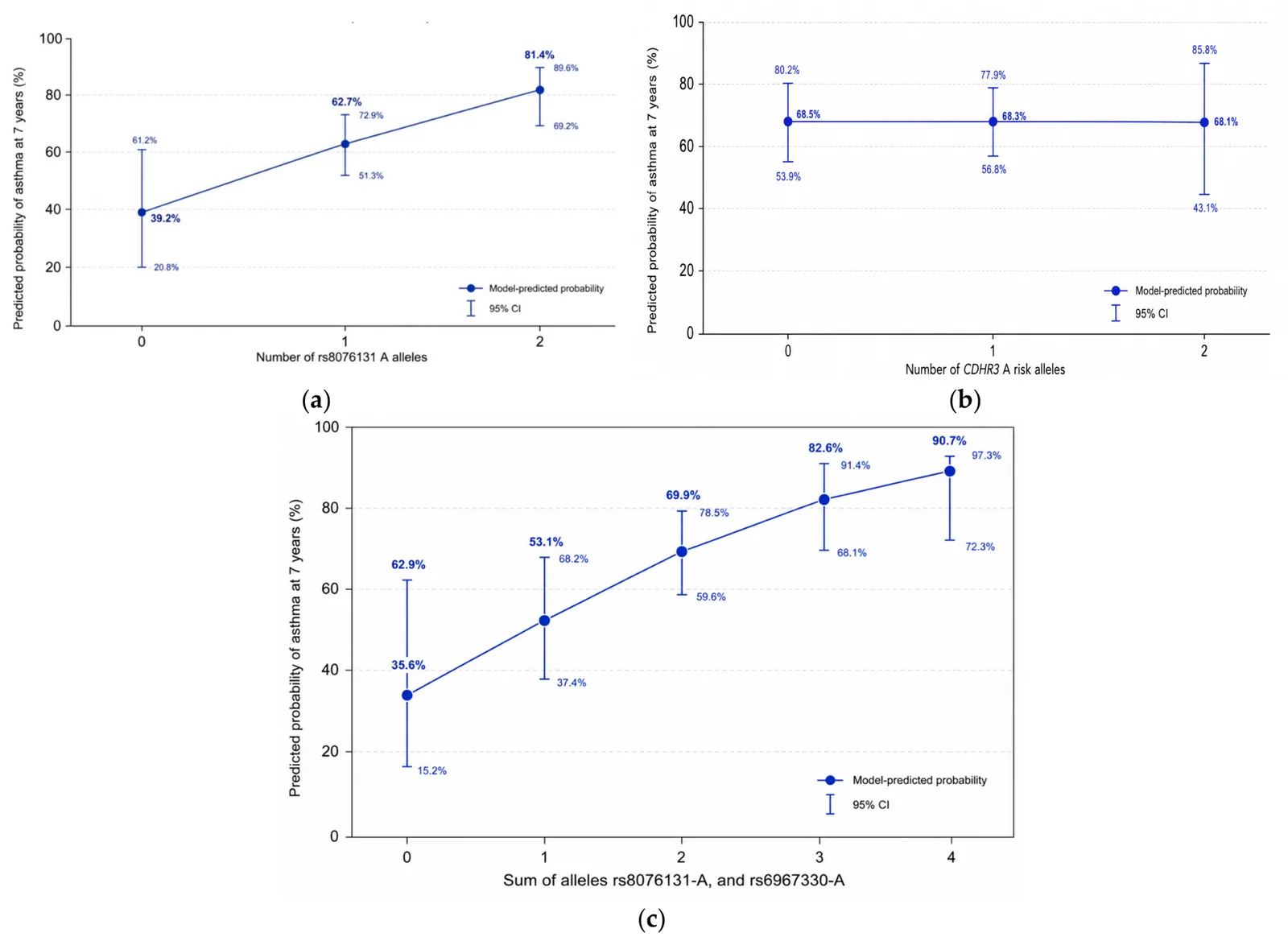
Predicted probability for asthma at 7 years in relation to; (**a**). the number of alleles rs8076131-A (*ORMDL3*) analyzed in N = 99 cases; OR = 2.61, 95% CI 1.38–4.94, *p* = 0.003. (**b**). The number of alleles A in rs6967330 (*CDHR3*) analyzed in N = 98 cases; OR = 0.99, 95% CI 0.49–1.99, *p* = 0.980. (**c**). The sum of alleles rs8076131-A, and rs6967330-A, analyzed in N = 98 cases; OR = 2.05, 95% CI 1.16–3.62, *p* = 0.013. Observed sample sizes: n = 1, 29, 40, 26, and 2 for 0–4 risk alleles, respectively. Points and connecting lines represent probabilities of asthma at 7 years predicted from an additive logistic regression model. Error bars represent the 95% confidence intervals (CIs) for the model-predicted probabilities. The number of rs8076131 A alleles, alleles A in rs6967330 (*CDHR3*) and the sum of alleles rs8076131-A, and rs6967330-A were included in the respective model as continuous additive genetic variables.

No association was found for rs6967330 and asthma at 7 years, but children carrying at least one A allele were more likely to have asthma at 7 years if they also were homozygotes rs8076131-AA, rs8079416-CC or rs3859192-TT (Table 4). The sum of the number of rs6967330-A and rs8076131-A alleles carried by an individual, was positively related to asthma at 7 years (OR = 2.05 (1.16–3.62), *p* = 0.013, Figure 3).

### 3.3. Associations Between Genetic Variants in the 17Q21 Locus and Medication

**Figure 4:**
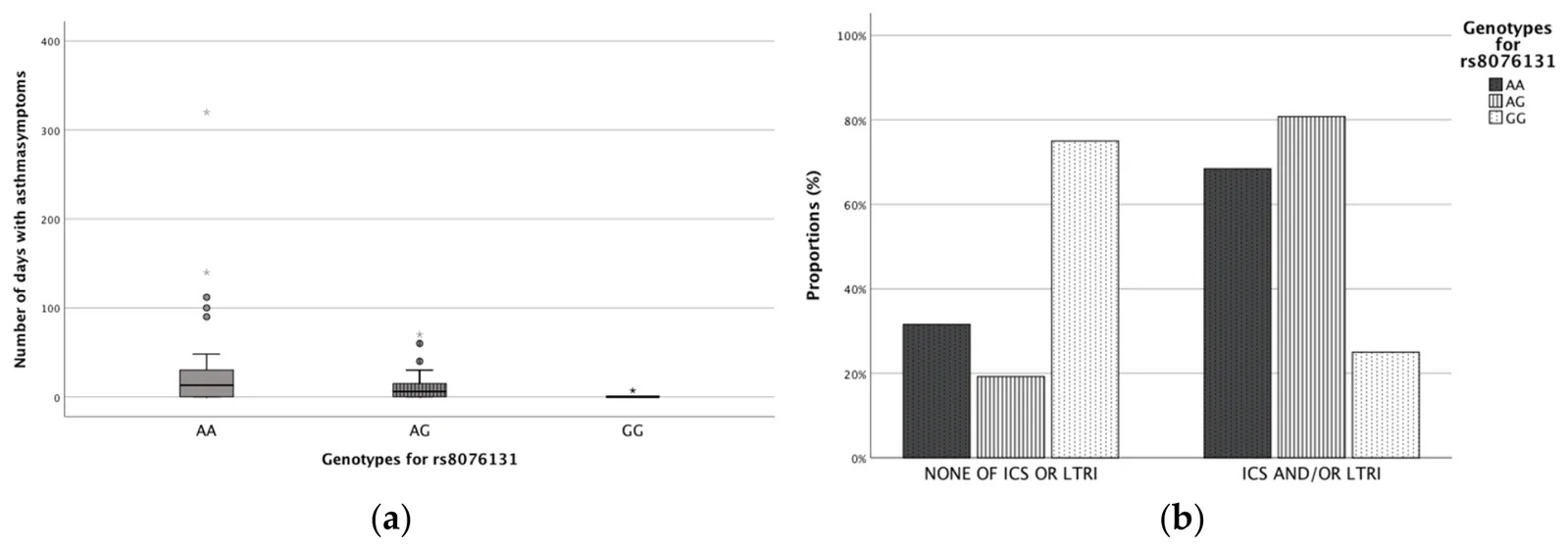
Association of rs8076131 genotypes with (**a**). number of days with asthma symptoms during the year prior to the follow-up at 7 years old (**AA**; median 13 days (IQR; 0–30), **AG**; median 6 days (IQR; 0–15), **GG**; median 0 days (IQR; 0–0). N = 66 children included in the analysis. Pairwise comparisons performed with Mann–Whitney U test; AA vs. GG; *p* = 0.011, AG vs. GG; *p* = 0.023), (symbols indicate outliers, defined as observations more than 1.5 × IQR below the first quartile or above the third quartile), and (**b**). use of inhaled corticosteroids (ICS) or leukotriene receptor antagonists (LTRAs) during the year prior to the follow-up at 7 years old (analysis performed with Chi-square test; AG vs. GG; OR = 12.6 (1.07–148.13), *p* = 0.048).

Rs8079416 (CC vs. CT; *p* = 0.046) and rs3859192 (TT vs. CT; *p* = 0.045, TT vs. CC; *p* = 0.032) were associated with the number of days with oral corticosteroids treatment during the first year after inclusion. Furthermore, rs8076131 was associated with the use of ICS or LTRA (AG vs. GG; OR = 12.6 (1.07–148.13), *p* = 0.048) as well as the number of days with asthma symptoms during the year prior to the follow-up at 7 years old (AA vs. GG; *p* = 0.011, AG vs. GG; *p* = 0.023, Figure 4).

## 4. Discussion

We focused on four genetic variants in the 17q21 locus: rs8076131 and rs12603332 located in the core LD block as well as rs8079416 and rs3859192 in the flanking region 17q21 locus. Rs8076131, a proxy for nine other variants in the high-LD region of 17q21 locus, was associated both with PW and asthma at 7 years. Rs12603332, although not related to PW, is shown to be significantly associated with asthma at 7 years among preschool wheezers. None of these four genetic variants is located in the coding regions in the 17q21 locus. Nevertheless, several studies support the importance of intronic variants for splicing efficiency or regulation of gene transcription [24,25]. While no association was found between rs8079416, rs3859192 (17q21 locus) or rs6967330 (*CDHR3*) and asthma at 7 years, subgroups of children with specific combinations of genotypes in these genetic variants had increased risk of asthma. Moreover, RV infection at inclusion was significantly related to asthma in strata of children with genotypes rs8079416-CC and rs3859192-TT.

Multiple genetic variants at the 17q21 locus have reproducibly been shown to be linked to childhood asthma [26] in ethnically diverse populations [25] but extensive linkage disequilibrium in this region has impeded identification of variants with causal association to asthma susceptibility. In children younger than 6 years with African American ancestry characterized by a shorter LD block at the 17q locus, the associations were scaled down to two genetic variants: rs2305480 and rs8076131 [27]. The association of the 17q21 locus with childhood asthma might be dependent on the time course of wheezing or differ among longitudinal wheezing phenotypes [28,29].

In our cohort, rs8076131-A was associated with both PW and asthma at 7 years whereas rs12603332-C was solely associated with increased risk of asthma at 7 years. Both of these genetic variants are located in the intronic region of *ORMDL3* and implicated in the expression of the same gene [30,31]. ORMDL3 is a transmembrane protein in the endoplasmic reticulum and regulates downstream pathways including sphingolipids, and may play a role in epithelial cell remodeling [32,33]. ORMDL3 is expressed in cell types involved in asthma pathogenesis [26]. In line with our results, Granell et al. have demonstrated varying associations of genetic variants in chromosome 17 with different wheezing phenotypes and shown that rs8076131-A is related to wheeze with onset from 18 months’ age and with persistent wheeze up to the age of 7.5 years in a cohort with central-European origin [30]. Ober et al. have shown a significant association of rs8076131-A with asthma in African American children (EVE cohort) [27], although in a study by Galanter et al., significance for this association in the same ethnic group was not reached [25].

Rs12603332-C is associated with increased *ORMDL3* expression in human immune cells [34]. E47, a transcription factor which is shown to inhibit the expression of several genes, binds in vitro to the rs12603332-T but does not bind to the rs12603332-C [34]. Rs12603332-T has been found to reduce the levels of *ORMDL3* mRNA in immune cells [34]. *ORMDL3* expression in peripheral blood leucocytes has been shown to be increased in children with asthma [35]. Therefore, there might be a biologically plausible link between rs12603332-C and asthma. Our findings are supported by a study by Sordillo et al. who highlighted the association of rs12603332-C with persistent wheeze throughout mid childhood but no clear relationship to transient wheeze in a multiethnic cohort recruited in USA [28]. Associations of genetic variants with asthma differ depending on age of onset and the severity of the disease [30,31,36,37]. Absence of association between rs12603332-C and PW in our cohort might be explained by a considerable proportion of preschool wheezers who had grown out of their symptoms by the age of 7 years, varying age at first wheezing episode or by an overall low power.

Our results point towards higher effect sizes of both rs8076131 and rs12603332 on asthma at 7 years compared to other studies [26]. This could be explained by our hospital-based cohort possibly being more clinically homogeneous and consisting of predominantly severe preschool wheeze with high prevalence of hospitalization at inclusion and a high risk of subsequent asthma at school age [38,39]. The modest odds ratios for the associations of the 17q21 locus with asthma, identified by GWAS, could be attributed to the clinical diversity of asthma, wide spectrum of age of onset or co-occurrence of other risk factors [31].

Rs8079416-C was associated with preschool wheeze but not with asthma at 7 years in the GEWAC cohort. Nor was there an association between rs3859192 and asthma. Rs8079416-C is shown to be related to asthma in a large study population including children with German and Austrian origin with a mean age of 11 years at inclusion [40,41], and in an adult cohort [42]. However, no significant association of the same genetic variant with asthma was found in a French cohort of individuals aged 6–65 years [36]. The rs8079416-C is associated with increased *ORMDL3* expression [30,41]. Rs3859192 (TT) is related to asthma in adults [42] but conflicting results are reported by studies in pediatric cohorts [19,36,40,43]. Rs3859192 is located in *GSDMA*, distal to the high-LD block of 17q locus and can have effects that are independent of genetic variants at the core region of 17q locus. It is shown to be a strong eQTL (expression Quantitative Trait locus) for *GSDMA* in lung tissue cells, correlated with lower expression of *GSDMA* [31]. However, *GSDMA* expression is found to be low in lung cells [31] and its association with asthma has not been established so far [31].

Among children with rs8079416-CC or rs3859192-TT, wheeze with RV infection at inclusion and rs6967330-AA or AG (*CDHR3*) genotypes were significantly associated with asthma at 7 years in our cohort. Effect alleles in the high-LD region of 17q21 locus are shown to confer increased asthma risk, especially in children who had RV-induced wheezing illness in early childhood [15]. Enhanced expression of *ORMDL3* may act in favor of viral replication in respiratory epithelial cells or even affect the repair of these cells after RV infection [15]. Rs8079416-C is associated with increased expression of *ORMDL3* [41] but there are, to our knowledge, no other previous reports about asthma-related combinations of genetic variants distally to the high-LD frame of 17q21 locus and rs6967330 (*CDHR3*).

While rs6967330-A (*CDHR3*) was related to PW in our cohort, as previously shown [44], the association of the same allele with asthma at 7 years was restricted to specific genotypes in the 17q21 locus. A significant association of rs6967330-A with asthma was genotype-specific for rs8076131-AA homozygotes. *CDHR3* is a gene known to code for the RV-C receptor, and is found to be related to early-life wheezing and asthma at school age by other studies [18,44]. The rs6967330-A (*CDHR3*) is shown to increase the expression of *CDHR3* as well as the risk of early-life wheezing episodes and their severity [17,38]. However, conflicting results are reported by a Finnish cohort [19]. The effect of rs6967330-A (*CDHR3*) on asthma is shown to be age-dependent, strongest among children with asthma onset until the age of 2 years, and modified by the *GSDMB* genotype, located in the high-LD region of 17q locus [39]. The role of *CDHR3* in the context of infections and asthma susceptibility is supposed to implicate the airway epithelium and is also found to be expressed in immune cells [39]. Similarly, *GSDMB* and *ORMDL3* also seem to play a functional role in immune cells [39]. In our cohort, a similar pattern of genotype-specific associations of rs6967330 (*CDHR3*) with asthma was expanded beyond the high-LD frame in the 17q21 locus as it was seen in rs3859192-TT (*GSDMA*) as well as rs8079416-CC homozygotes (*LRRC3C*).

Strengths of this study were the extensive phenotypical characterization of the recruited preschool wheezers and the longitudinal design that allowed analyses of multiple associations at different age points. Some associations were shown to differ between preschool age and the age of 7 years. Another asset is the hospital-based enrolment of the cohort, thus minimizing misclassification of outcomes. The linkage disequilibrium in the region is high, and we have focused on four genetic variants, but their effects might still be at least partly dependent on each other. There is a possibility that the lack of significance for associations of rs8079416, or rs3859192 or other variants with asthma is due to limited sample size. However, the relatively high prevalence of doctor’s asthma diagnosis at preschool age and of asthma at 7 years in GEWAC reflects a more severe wheezing phenotype, thereby increasing the chance of detecting associations and interactions [38,39] despite their relatively low effect sizes.

The relatively small sample size, particularly within stratified subgroups, resulted in wide confidence intervals for some of the estimated associations. Accordingly, the large effect estimates observed in certain subgroups should be interpreted with caution, as they may partly reflect unstable subgroup estimates. In addition, the possibility of false-positive findings due to multiple testing cannot be excluded. This is particularly relevant for associations with borderline statistical significance or very wide 95% confidence intervals. Therefore, these findings should be interpreted cautiously and require confirmation in larger, independent cohorts. The effect sizes observed in our study should also be considered in the context of the more modest effect sizes reported in large genome-wide association studies of the 17q21 locus. Moreover, our cohort was a selected hospital-based population and may therefore represent children with more severe or persistent wheezing phenotypes rather than the broader spectrum of wheezing and asthma in the community, limiting the generalizability of the findings.

The stratified analyses identified patterns suggesting that the associations between specific genetic variants and asthma may differ according to clinical or environmental characteristics, such as rhinovirus infection. However, logistic regression models including interaction terms (genotype for rs3859192 and rs8079416 × rhinovirus infection) should be performed in larger groups to provide proof of interaction. Therefore, the observed patterns suggest potential effect modification that warrants further investigation. Larger studies with adequate power for interaction testing are needed to determine whether these associations represent true gene–environment or gene–gene interactions. An additional limitation is the time elapsed after cohort recruitment and statistical analysis. Although the cohort was recruited during an earlier period (2008–2012), the genetic variants investigated represent stable inherited characteristics, and the prospective longitudinal design remains relevant to understanding the relationship between early-life wheezing, genetic susceptibility and subsequent asthma development. The specific role of the 17q21 locus in asthma development following early-life wheezing remains of biological and clinical interest.

Despite these limitations, the present findings provide informative, potentially hypothesis-generating observations regarding the relationship between genetic susceptibility and early-life clinical exposures in children with wheezing. The consistency of some associations across clinically relevant subgroups, together with the biological plausibility of the 17q21 locus and *CDHR3* in childhood respiratory disease, supports further investigation in larger and more diverse cohorts.

## 5. Conclusions

In our longitudinal, hospital-based cohort we could replicate associations of genetic variants in the 17q21 locus with preschool wheeze and school-age asthma. Some of these associations might be phenotype- or age-specific. Our study highlights the importance of asthma heritability by reporting higher magnitudes of associations than reported before, possibly owing to the homogeneity and severity of wheezing phenotypes included. Our findings suggest previously unreported patterns of association between genetic variants in the 17q21 locus, *CDHR3*-rs6967330 and asthma according to rhinovirus exposure. Future studies with larger sample sizes are warranted to investigate these potential interactions.

## Figures and Tables

**Table 1:** Inclusion and exclusion for cases and controls enrolled in GEWAC cohort.

	Inclusion Criteria	Exclusion Criteria
Cases N = 156	- Age of 6 to 48 months - Acute symptoms of wheeze	- Prematurity (birth before 36 gestational weeks) - Chronic disease - Simultaneous complications (e.g., Sepsis, bacterial pneumonia)
Controls N = 102	- Age of 6 to 48 months	- Prematurity (birth before 36 gestational weeks) - History of bronchial obstruction and asthma - Known sensitization to airborne allergens.

**Table 2:** Associations of genotypes in studied genetic variants with preschool wheeze (statistical analyses performed in cases (Ν = 123–125) and healthy controls (N = 94–96) who could be genotyped for the respective genetic variant) and asthma at 7 years (cases without (N = 30–31) and with asthma (N = 67–68) included in the analyses).

Genetic Variant	Genotypes	Associations with Preschool Wheeze		Associations with Asthma at 7 Years	
		OR (95% CI)	*p* Value	OR (95% CI)	*p* Value
Rs9303277	CC vs. CT	2.67 (1.38–5.18)	**0.003**	1.54 (0.57–4.15)	0.388
	CC vs. TT	3.217 (1.47–7.04)	**0.003**	4.43 (1.29–15.23)	**0.015**
	CT vs. TT	1.2 (0.61–2.34)	0.594	2.87 (0.88–9.38)	0.076
Rs4795397	AA vs. AG	2.77 (1.48–5.18)	**0.001**	2.06 (0.78–5.43)	0.141
	AA vs. GG	3.59 (1.63–7.90)	**0.001**	4.43 (1.22–16.17)	**0.033**
	AG vs. GG	1.30 (0.62–2.73)	0.495	2.15 (0.60–7.77)	0.236
Rs12936231	CC vs. CG	2.47 (1.27–4.80)	**0.007**	1.50 (0.56–4.01)	0.425
	CC vs. GG	3.50 (1.60–7.68)	**0.001**	3.94 (1.12–13.83)	**0.047**
	CG vs. GG	1.42 (0.71–2.82)	0.321	2.64 (0.79–8.80)	0.109
Rs8067378	AA vs. AG	2.51 (1.30–4.90)	**0.006**	1.49 (0.56–4.01)	0.425
	AA vs. GG	3.27 (1.40–7.15)	**0.002**	4.43 (1.29–15.23)	**0.015**
	AG vs. GG	1.30 (0.66–2.57)	0.450	2.97 (0.91–9.69)	0.066
Rs2305480	CC vs. CT	2.81 (1.51–5.24)	**0.001**	1.90 (0.73–4.98)	0.189
	CC vs. TT	3.88 (1.75–8.60)	**0.001**	7.60 (1.90–30.44)	**0.004**
	CT vs. TT	1.38 (0.65–2.93)	0.402	4.00 (1.01–15.78)	0.051
Rs7216389	TT vs. CT	2.50 (1.32–4.76)	**0.005**	1.32 (0.50–3.50)	0.576
	TT vs. CC	3.25 (1.49–7.11)	**0.003**	5.94 (1.62–21.84)	**0.008**
	CT vs. CC	1.30 (0.64–2.62)	0.465	4.50 (1.25–16.22)	**0.017**
Rs4065275	GG vs. AG	2.53 (1.32–4.86)	**0.005**	1.38 (0.51–3.74)	0.529
	GG vs. AA	2.80 (1.29–6.09)	**0.008**	4.92 (1.46–16.64)	**0.008**
	AG vs. AA	1.11 (0.56–2.19)	0.769	3.57 (1.10–11.57)	**0.030**
Rs8076131	AA vs. AG	2.75 (1.48–5.11)	**0.001**	1.75 (0.66–4.66)	0.257
	AA vs. GG	3.50 (1.60–7.66)	**0.001**	8.55 (2.18–33.59)	**0.002**
	AG vs. GG	1.27 (0.60–2.68)	0.529	4.88 (1.25–19.03)	**0.017**
Rs12603332	CC vs. CT	1.71 (0.91–3.21)	0.093	1.38 (0.50–3.80)	0.534
	CC vs. TT	1.91 (0.88–4.16)	0.100	4.50 (1.27–15.90)	**0.016**
	CT vs. TT	1.12 (0.55–2.25)	0.756	3.26 (1.01–10.60)	**0.044**
Rs17608925	TT vs. CT	2.64 (1.16–6.03)	**0.018**	3.15 (0.78–12.70)	0.130
	TT vs. CC	0.73 (0.07–8.23)	0.801	2.52 (0.15–41.87)	0.497
	CT vs. CC	0.28 (0.02–3.46)	0.543	0.80 (0.04–17.20)	1.000
Rs4795405	CC vs. CT	2.54 (1.37–4.71)	**0.003**	1.92 (0.74–4.97)	0.176
	CC vs. TT	3.73 (1.66–8.37)	**0.001**	6.48 (1.58–26.61)	**0.010**
	CT vs. TT	1.47 (0.68–3.16)	0.326	3.38 (0.84–13.52)	0.095
Rs8079416	CC vs. CT	1.52 (0.78–2.97)	0.214	1.05 (0.39–2.83)	0.920
	CC vs. TT	2.42 (1.12–5.23)	**0.024**	1.86 (0.56–6.13)	0.306
	CT vs. TT	1.59 (0.82–3.06)	0.169	1.77 (0.59–5.32)	0.309
Rs3859192	TT vs. CT	1.82 (0.94–3.53)	0.076	1.05 (0.38–2.90)	0.931
	TT vs. CC	1.92 (0.92–4.01)	0.082	1.17 (0.38–3.57)	0.787
	CT vs. CC	1.06 (0.56–2.00)	0.870	1.12 (0.40–3.14)	0.836

Bold values indicate statistically significant associations (*p* < 0.05).

**Table 3:** The relationship between genetic variants in the 17q21 locus (genotypes)/number of effect alleles (additive model) and preschool wheeze (PW) as well as asthma at 7 years when adjusting for other significant factors for PW and for asthma at 7 years.

Genetic Variant	Genotypes	Associations with Preschool Wheeze		Associations with Asthma at 7 Years	
		OR (95% CI)	*p* Value	OR (95% CI)	*p* Value
Rs8076131	AA vs. GG	4.91 (1.88–12.80)	**0.001**	8.50 (1.78–40.68)	**0.007**
	AG vs. GG	1.50 (0.69–3.74)	0.388	4.08 (0.86–19.51)	0.078
	GG = 0 AG = 1 AA = 2	2.36 (1.47–3.78)	**<0.001**	2.69 (1.31–5.51)	**0.007**
Rs12603332	CC vs. TT	2.26 (0.88–5.81)	0.092	7.17 (1.45–35.56)	**0.016**
	CT vs. TT	0.91 (0.39–2.10)	0.816	4.89 (1.07–22.36)	**0.041**
	TT = 0 CT = 1 CC = 2	1.55 (0.97–2.48)	0.066	2.37 (1.12–5.03)	**0.025**
Rs8079416	CC vs. TT	3.18 (1.26–8.05)	**0.015**	1.81 (0.47–6.99)	0.387
	CT vs. TT	2.05 (0.923–4.56)	0.078	1.39 (0.39–4.92)	0.609
	TT = 0 CT = 1 CC = 2	1.78 (1.12–2.84)	**0.015**	1.34 (0.69–2.63)	0.388
Rs3859192	TT vs. CC	0.45 (0.18–1.09)	0.078	0.75 (0.21–2.68)	0.655
	CT vs. CC	0.52 (0.23–1.16)	0.111	0.97 (0.31–3.01)	0.954
	CC = 0 CT = 1 TT = 2	0.68 (0.43–1.05)	0.083	0.87 (0.46–1.65)	0.662

Bold values indicate statistically significant associations (*p* < 0.05). Adjusted ORs calculated by logistic regression in multivariate models including factors associated with preschool wheeze (gender, eczema at inclusion, smoking during pregnancy, childcare or family home attendance, reported RSV infection, heredity for asthma and for allergy in parents) and asthma at 7 years (RV infection at inclusion and current allergic rhinitis or conjunctivitis).

**Table 4:** Relationship between rs6967330 and asthma at 7 years in the whole study population (**a**) and in strata defined by genotypes for genetic variants in 17q21 locus (**b**). Children with rs6967330-AA or AG had greater risk for asthma at 7 years than homozygotes GG if they also carried specific genotypes in 17q21 locus.

(a)																		
Association Between rs6967330 in *CDHR3* and Asthma at 7 Years																		
Asthma at 7 Years		AA N (%)	AG N (%)		GG N (%)	AA vs. AG			AA vs. GG			AG vs. GG				AA/AG vs. GG		
						*p* Value	OR		*p* Value	OR		*p* Value		OR		*p* Value		OR
Non-asthmatics		4 (57.1)	12 (24.5)		15 (35.7)	0.094	0.24 (0.05–1.24)		0.407	0.42 (0.08–2.12)		0.243		1.71 (0.69–4.24)		0.452		1.39 (0.59–3.27)
Asthmatics		3 (42.9)	37 (75.5)		27 (64.3)													
(**b**)																		
**Association Between rs6967330 in *CDHR3* and Asthma at 7 Years**																		
**Genetic Variants** **17q21 Locus**		**AA vs. AG**				**AA vs. GG**					**AG vs. GG**				**AA/AG vs. GG**			
		***p* Value**		**OR**		***p* Value**		**OR**			***p* Value**		**OR**		***p* Value**		**OR**	
Rs8076131	AA	0.151		0.04 (0.00–1.33)		1.000		0.62 (0.03–11.28)			**0.007**		14.15 (1.59–126.12)		**0.028**		7.39 (1.36–40.03)	
	AG	1.000		0.46 (0.023–8.83)		1.000		0.43 (0.02–8.04)			1.000		0.94 (0.23–3.92)		1.000		0.86 (0.22–3.37)	
	GG	1.000		1.00 (0.06–15.99)		1.000		--- *			1.000		--- *		1.000		--- *	
Rs12603332	CC	1.000		--- *		0.529		--- *			0.118		4.50 (0.75–26.93)		0.105		5.10 (0.86–30.27)	
	CT	0.083		--- *		0.093		--- *			0.862		1.13 (0.29–4.44)		0.743		0.81 (0.22–2.92)	
	TT	1.000		0.42 (0.03–6.06)		1.000		--- *			0.462		--- *		0.475		--- *	
Rs8079416	CC	1.000		--- *		0.462		--- *			**0.012**		11.90 (1.85–76.53)		**0.006**		12.60 (1.97–80.76)	
	CT	0.247		0.19 (0.01–2.62)		0.220		0.18 (0.01–2.36)			1.000		0.96 (0.25–3.68)		0.633		0.74 (0.21–2.59)	
	TT	0.569		3.58 (0.03–5.11)		0.486		0.17 (0.01–4.51)			1.000		0.47 (0.04–5.90)		0.603		0.38 (0.03–4.55)	
Rs3859192	TT	1.000		--- *		1.000		--- *			**0.042**		6.40 (1.16–35.44)		**0.042**		6.40 (1.16–35.44)	
	CT	**0.021**		--- *		0.052		--- *			0.469		2.02 (0.43–9.46)		0.987		1.01 (0.27–3.78)	
	CC	1.000		2.25 (0.19–27.37)		1.000		0.86 (0.05–13.45)			0.400		0.38 (0.06–2.53)		0.667		0.45 (0.07–2.81)	

--- * OR not possible to calculate. Bold values indicate statistically significant associations (*p* < 0.05).

## Data Availability

Due to the nature of this research, participants of this study did not agree for their data to be shared publicly, so supporting data are not available.

## Supplementary Materials

The following supporting information can be downloaded at https://www.mdpi.com/article/10.3390/children13091188/s1. Table S1: Genetic variants included in the study; Table S2: Baseline characteristics in cases and controls. Clinical as well as laboratory parameters at inclusion and 7 years in cases with and without asthma at 7 years. Table S3: Drop-out analysis of cases lost to follow-up. Table S4: Relationship between rs6967330 and preschool wheeze in the whole study population (a) and in strata defined by genotypes for genetic variants in 17q21 locus (b).

## Abbreviations

The following abbreviations are used in this manuscript:

aOR	Adjusted odds ratio
*CDHR3*	Cadherin-related family member 3
GEWAC	Gene Expression in Wheezing and Asthmatic Children
*GSDMA*	Gasdermin A
*GSDMB*	Gasdermin B
GEWAS	Genome-wide association studies
eQTL	Expression Quantitative Trait locus
ICS	Inhaled corticosteroids
*IKZF*3	IKAROS Family Zinc Finger 3
LD	Linkage disequilibrium
*LRRC3C*	Leucine Rich Repeat Containing 3C
LTRA	Leukotriene receptor antagonist
*MED24*	Mediator Complex Subunit 24
*ORMDL3*	Orosomucoid-like 3
PW	Preschool wheeze
RSV	Respiratory syncytial virus
RV	Rhinovirus
*ZPBP2*	Zona Pellucida Binding Protein 2
