# Supplementary material for: Associations Between Genetic Variants in the 17q21 Locus, CDHR3 and Childhood Asthma
Source: Children (Basel). 2026 Sep 3;13(9):1188. doi: 10.3390/children13091188 (PMC13605119; doi:10.3390/children13091188)
Supplement: Supplementary file 1 [file children-13-01188-s001.zip › children-4450346-supplementary.pdf]

Table S1. Genetic variants included in the study.
Source: https://www.ensembl.org/search

**Genetic variant**	**Gene**	**Position** (GRCh38)	**Location**	**Alleles** ancestral/derived **Associated allele**
Rs9303277	*IKZF3*	chr17:39820216	Intron variant	T/**C**
Rs4795397	*ZPBP2*	chr17:39867492	Regulatory region variant	**A**/G
Rs12936231	*ZPBP2*	chr17:39872867	Intron variant	G/**C**
Rs8067378	*GSDMB*	chr17:39895095	Regulatory region variant	G/**A**
Rs2305480	*GSDMB*	chr17:39905943	Missense variant	G/**T**
Rs7216389	*GSDMB*	chr17:39913696	Intron 1	**T**/C
Rs4065275	*ORMDL3*	chr 17:39924612	Intron variant	A/**G**
Rs8076131	*ORMDL3*	chr17:39924659	Intron variant	**A**/G
Rs12603332	*LRRC3C/ ORMDL3*	chr17:39926554	5 prime UTR variant/ Intron variant	T/**C**
Rs17608925	*LRRC3C/ ORMDL3*	chr17:39926578	5 prime UTR variant/ Intron variant	**T**/C
Rs4795405	*LRRC3C*	chr17:39932164	Intron variant	T/**C**
Rs8079416	*LRRC3C*	chr17:39936460	Intron variant	T/**C**
Rs3859192	*GSDMA*	chr17:39972395	Intron variant	C/**T**
Rs6967330	*CDHR3*	chr7:106018005	Missense variant	**A**/G

Table S2. Baseline characteristics in cases and controls. Clinical as well as laboratory parameters at inclusion and 7 years in cases with and without asthma at 7 years.

	Controls at inclusion N=96	Cases at inclusion N=125	P value Preschool wheeze	Cases without asthma at 7 years N= 31/99 (31.3%)	Cases with asthma at 7 years N= 68/99 (68.7%)	P value Asthma at 7 years
**Mean age (range) at inclusion (months)**	20.6 (6-45)	19.2 (6-42)	0.305	17.7 (8-37)	19.5 (6-42)	0.326
**Male, %(n)**	78.1 (75)	64.0 (80)	**0.023**	71 (22)	60.3 (41)	0.306
**Heredity asthma parents, %(n) [a]**	13.2 (12)	35.5 (44)	**<0.001**	22.6 (7)	40.3 (27)	0.087
**Heredity allergy parents, %(n) [a]**	43.0 (40)	67.7 (84)	**<0.001**	64.5 (20)	71.6 (48)	0.477
**Mother Caucasian %(n) [b]**	76.4 (68)	81.6 (102)	0.354	83.9 (26)	76.5 (52)	0.404
**Father Caucasian %(n) [b]**	76.1 (67)	84.7 (105)	0.117	83.9 (26)	85.1 (57)	0.878
**Exclusive breastfeeding at least 4 months, %(n) [a]**	68.8 (64)	57.9 (70)	0.100	67.7 (21)	58.5 (38)	0.382
**Attend childcare or family home care, %(n) [a]**	52.7 (49)	71.8 (89)	**0.004**	77.4 (24)	73.1 (49)	0.651
**Smoking during pregnancy, %(n) [a]**	3.2 (3)	10.4 (13)	**0.042**	3.2 (1)	11.8 (8)	0.266
**Furred pets at home, %(n) [a]**	44.6 (41)	50.8 (63)	0.364	58.1 (18)	41.8 (28)	0.133
**Reported RS infection, %(n) [a]**	4.3 (4)	28.0 (35)	**<0.001**	25.8 (8)	26.5 (18)	0.944
**RV at inclusion, %(n) [a]**	-	-	-	23.3 (7)	47.8 (32)	**0.023**
**Eczema at inclusion, %(n) [a]**	7.4 (7)	20.8 (26)	**0.006**	19.4 (6)	23.5 (16)	0.643
**Current allergic rhinitis or conjunctivitis, %(n) [a]**	0.0 (0)	0.01 (1)	1.000	0.0 (0)	22.4 (15)	**0.004**
**Fx5 positive at preschool age, %(n) [b]**	17.9 (14)	23.8 (29)	0.328	26.7 (8)	19.4 (13)	0.422
**Phadiatop positive at preschool age, %(n) [b]**	3.8 (3)	9.8 (12)	0.117	3.3 (1)	13.4 (9)	0.167
**Fx5 positive at 7 years, %(n)[c]**	36.0 (9)	22.6 (19)	0.179	13.0 (3)	26.2 (16)	0.198
**Phadiatop positive at 7 years, %(n) [c]**	8.0 (2)	28.6 (24)	**0.034**	26.1 (6)	42.6 (26)	0.164
**Vitamin D insufficiency at baseline [b]**	24.5 (23)	36.9 (41)	0.055	27.6 (8)	39.0 (23)	0.293
**Vitamin D insufficiency at 7 years [c]**	84.0 (21)	89.3 (75)	0.490	87.0 (20)	90.2 (55)	0.700

[a] 91-94 healthy controls/119-125 cases at inclusion/ 29-31 cases without asthma at 7 years/65-68 cases with asthma at 7 years.

[b] 78-94 healthy controls/111-122 cases at inclusion/ 29-30 cases without asthma at 7 years/59-67 cases with asthma at 7 years.
[c] 25 healthy controls/84 cases at inclusion/ 23 cases without asthma at 7 years/61 cases with asthma at 7 years.
Bold values indicate statistically significant associations (P < 0.05)

Table S3. Drop-out analysis of cases lost to follow-up.

	Cases included in analyses at 7 years<br><br>N=99	Cases lost to follow-up<br><br>N=26	P value
**Mean age (range) at inclusion (months)**	18.9 (6.0-42.0)	20.2 (6.0-41.0)	0.549
**Male, %(n)**	63.6 (63)	65.4 (17)	0.869
**Heredity asthma parents, %(n)** [a]	34.7 (34)	38.5 (10)	
**Heredity allergy parents, %(n)** [a]	69.4 (68)	61.5 (16)	0.447
**Exclusive breastfeeding at least 4 months, %(n)** [a, b]	61.5 (59)	44 (11)	0.115
**Attend childcare or family home care, %(n)** [a]	74.5 (73)	61.5 (16)	0.192
**Smoking during pregnancy, %(n)**	9.1 (9)	15.4 (4)	0.349
**Furred pets at home, %(n)** [a]	46.9 (46)	65.4 (17)	0.094
**Reported RS infection, %(n)**	26.3 (26)	34.6 (9)	0.399
**RV at inclusion, %(n)** [a]	40.2 (39)	53.8 (14)	0.212
**Eczema at inclusion, %(n)**	22.2 (22)	15.4 (4)	0.445
**Current allergic rhinitis or conjunctivitis, %(n)** [a, b]	0.0 (0)	4 (1)	0,052
**Fx5 positive at preschool age, %(n)** [a, b]	21.6 (21)	32.0 (8)	0.278
**Phadiatop positive at preschool age, %(n)** [a, b]	10.3 (10)	8.0 (2)	0.730
**Vitamin D insufficiency at baseline** [b, c]	35.2 (31)	43.5 (10)	0.465

[a] 94-98 cases included in the study
[b] 23-25 cases lost to follow-up
[c] 88 cases included in the study

Table S4. Relationship between rs6967330 and preschool wheeze in the whole study population **(4a)** and in strata defined by genotypes for genetic variants in 17q21 locus **(4b)**.

	Association CDHR3 genetic variants with **Preschool wheeze**											
Affection status	**AA** (N) %	**AG** (N) %	**GG** (N) %	P value	**AA vs AG**		**AA vs GG**		**AG vs GG**		**AA/AG vs GG**	
					P value	OR	P value	OR	P value	OR	P value	OR
**Cases**	8 (80.0)	65 (68.4)	51 (45.1)	**0.001**	0.720	1.85 (0.37-9.22)	**0.047**	**4.86** (0.99-23.92)	**0.001**	**2.63** (1.49-4.66)	**<0.001**	**2.77** (1.59-4.84)
**Controls**	2 (20.0)	30 (31.9)	62 (54.9)									

S4a.

		Association CDHR3 genetic variants with **Preschool wheeze**											
Stratified by		**AA** (N)*	**AG** (N)	**GG** (N)	P value	**AA vs AG**		**AA vs GG**		**AG vs GG**		**AA/AG vs GG**	
						P value	OR	P value	OR	P value	OR	P value	OR
**Rs8076131**	AA	3	41	41	0.079	1.000	---*	0.290	---*	0.052	2.64 (0.98-7.14)	**0.033**	2.88 (1.07-7.75)
	AG	4	35	54	0.276	1.000	0.67 (0.08-5.30)	1.000	1.35 (0.18-10.29)	0.109	2.02 (0.85-4.80)	0.144	1.94 (0.84-4.47)
	GG	3	19	18	**0.004**	0.273	---*	**0.015**	---*	**0.010**	6.88 (1.48-32.01)	**0.003**	8.75 (1.93-39.75)
**Rs12603332**	CC	2	29	35	0.113	1.000	---*	0.495	---*	0.073	2.65 (0.90-7.79)	**0.049**	2.89 (0.987-8.44)
	CT	5	39	62	**0.040**	0.647	0.67 (0.10-4.52)	0.650	1.94 (0.30-12.47)	**0.012**	2.92 (1.25-6.79)	**0.012**	2.78 (1.24-6.24)
	TT	3	26	15	**0.032**	0.268	---*	**0.043**	----	0.055	3.75 (0.94-14.96)	**0.026**	4.50 (1.15-17.68)
**Rs8079416**	CC	1	26	30	**0.003**	1.000	---*	0.484	---*	**0.001**	8.76 (2.16-35.56)	**0.001**	9.14 (2.26-37.01)
	CT	5	47	56	0.479	0.637	2.71 (0.28-26.21)	0.371	3.47 (0.36-33.00)	0.541	1.28 (0.58-2.80)	0.403	1.39 (0.64-2.99)
	TT	4	22	27	**0.014**	1.000	1.46 (0.24-8.67)	0.087	8.57 (0.76-96.52)	**0.008**	5.00 (1.47-16.99)	**0.004**	5.40 (1.66-17.57)
	TT	1	33	27	0.068	1.000	---*	1.000	---*	**0.028**	3.45	**0.023**	3.58

**Rs3859192**											(1.12-10.63)		(1.17-11.01)
	CT	3	39	55	0.080	0.541	---*	0.106	0.46 (0.34-0.61)	0.124	1.92 (0.83-4.43)	0.065	2.16 (0.95-4.93)
	CC	6	23	31	0.116	1.000	1.07 (0.16-7.15)	0.371	3.17 (0.50-20.04)	0.054	2.97 (0.97-9.12)	**0.038**	3.01 (1.05-8.62)

S4b.

---*OR not possible to calculate.
